# The ventilatory response to arousal from sleep is elevated in young individuals with post-traumatic stress disorder symptoms

**DOI:** 10.1007/s44470-026-00050-6

**Published:** 2026-05-07

**Authors:** Maya T. Schenker, Amber Russell, Darsh Cherian, Yuhe Pan, Gemma Bruce, Lilith Zeng, Joanne Avraam, Fergal J. O’Donoghue, Kim L. Felmingham, Amy S. Jordan

**Affiliations:** 1 Melbourne School of Psychological Sciences, The University of Melbourne, Grattan St, Parkville, VIC 3010 Australia; 2 The Institute for Breathing and Sleep, Austin Health, Heidelberg, VIC Australia; 3 Faculty of Medicine, Dentistry and Health Sciences, The University of Melbourne, Parkville, VIC Australia

**Keywords:** Obstructive sleep apnea, Trauma exposure, Pathophysiology, Sleep

## Abstract

**Purpose:**

Obstructive sleep apnea (OSA) is reported to be more prevalent in posttraumatic stress disorder (PTSD) compared to the general population for unknown reasons. Most prior studies have assessed military populations who commonly possess other OSA risk factors. This study aimed to determine whether: 1) OSA prevalence is increased in young adults with PTSD symptoms exposed to non-military traumas; and 2) the ventilatory response to arousal from sleep - an OSA pathogenic trait - differs according to PTSD symptoms.

**Methods:**

Individuals with a range of PTSD symptoms (PTSD symptom checklist for DSM-5, PCL-5) completed an overnight sleep study. OSA prevalence and the ventilatory response to brief spontaneous arousals were compared between those with Likely PTSD (trauma exposure, intrusions and PCL-5 > 33), Subsyndromal PTSD (trauma exposure, intrusions and PCL-5 15-33) and No PTSD (PCL-5 < 15).

**Results:**

Data were obtained in 60 individuals, 18 with Likely PTSD, 19 Subsyndromal PTSD and 23 No PTSD. The number found to have OSA (4 in Likely PTSD, 3 in Subsyndromal PTSD and 1 in No PTSD, *p* = .21) and the mean apnea-hypopnea index did not differ between groups (p < .98). The ventilatory response to arousal was assessed in 29 individuals without OSA who had good nasal pressure traces and was significantly larger in individuals with Likely PTSD and subsyndromal PTSD than No PTSD (*p* < .001).

**Conclusions:**

Although these young individuals with Likely PTSD did not commonly have OSA, the ventilatory response to arousal was elevated, which when combined with other OSA risk factors may predispose to OSA.

**Brief summary:**

**Current Knowledge/Study Rationale:**

Obstructive sleep apnea has been reported to be much more common in military veterans with posttraumatic stress disorder (PTSD) than in the general population. Whether this is a result of factors specific to military veterans or due to other reasons was unknown.

**Study Impact:**

This study found that young adults with non-military trauma exposure and likely PTSD did not have elevated rates of OSA. However, their ventilatory response to brief spontaneous arousal from sleep was elevated, which may contribute to OSA development when other risk factors are present (older age, obesity etc) and may also represent a PTSD-specific OSA treatment pathway.

## Introduction

Posttraumatic Stress Disorder (PTSD) is a persistent, costly and often debilitating mental health condition that develops following exposure to a traumatic event. Sleep disturbances are pervasive in PTSD, with up to 90% of patients reporting insomnia and/or nightmares [1]. These disturbances perpetuate PTSD symptoms and hinder treatment responses [2], suggesting that improving sleep may be a pathway to enhancing PTSD treatment.

In addition to insomnia and nightmares, obstructive sleep apnea (OSA) appears to occur at a much higher rate in PTSD than in the general population, with 75.7% of people with PTSD being reported to have at least mild OSA and 43.6% at least moderate OSA [3]. However, many of the prior studies have investigated military veterans who commonly have other risk factors for OSA such as being male, overweight, older age and having high rates of substance use disorders [3, 4]. There are relatively few prior studies in non-military populations without these comorbidities and whether OSA prevalence is increased or not is unclear as findings are mixed [5–8].

The reason why OSA is more common in some PTSD populations is also currently unknown. Four traits are thought to be the main contributors to OSA in the general population: a highly collapsible upper airway, poor ability of upper airway muscles to dilate the airway, a low level of respiratory effort that elicits arousal from sleep, and an unstable respiratory control system [9]. While there are plausible reasons why these four traits may differ in PTSD, current evidence suggests that they do not differ between veterans with both PTSD and OSA, as compared to veterans with OSA but without PTSD [10]. Thus, PTSD and OSA may commonly co-occur for other reasons.

We have previously shown that individuals with PTSD have increased perception of breathlessness during wakefulness [11], potentially due to greater interoceptive sensitivity [12]. Similarly, individuals with PTSD have also been shown to have greater emotional responsiveness to elevations in inspired carbon dioxide (CO_2_) [13, 14]. If individuals experience heightened CO_2_ responsiveness and breathlessness during brief arousals, this could lead to an elevated ventilatory response to arousal from sleep, another factor that appears to contribute to OSA development [15]. Thus, the aims of this study were to 1) investigate the prevalence of OSA in a younger adult, non-military, mixed-trauma population with PTSD symptoms, and 2) compare the magnitude of the ventilatory response to arousal from sleep in individuals with and without PTSD symptoms. In addition to studying Likely PTSD and No PSTD groups, we also chose to investigate participants with subsyndromal PTSD because many individuals experience symptoms not meeting full PTSD diagnostic criteria following trauma exposure [16], and such individuals have employment problems, social disruptions and lower quality of life compared to people with no symptoms [17, 18], yet whether they have an increased prevalence of OSA is unknown. Understanding the relationship between OSA and PTSD is important because individuals with both OSA and PTSD have worse symptoms and higher healthcare utilisation than individuals with either condition alone [7, 19]. In addition, the gold standard treatment for OSA - continuous positive airway pressure (CPAP) - is only used in <30% of PTSD patients due to anxiety and claustrophobia [19, 20] and so identifying alternative treatments based on PTSD specific OSA pathophysiology could be highly beneficial.

## Methods

### Participants

Participants aged 18–65 years were recruited from the University of Melbourne community via the Research Experience Program for first-year psychology students as well as advertisements placed around the Parkville campus and online noticeboards. Participants recruited from the Research Experience Program were reimbursed with course credit upon completing the online screening survey and all participants that completed the sleep study component were reimbursed $100. The study was approved by the University of Melbourne Human Research Ethics Committee (#23625) and the study was performed in accordance with the ethical standards as laid down in the 1964 Declaration of Helsinki and its later amendments. The data underlying this article cannot be shared publicly because extended consent was obtained from participants, such that the data can only be used for this project or closely related projects. The data will be shared on reasonable request to the corresponding author if this criteria is met.

Recruitment and data collection occurred in two stages: a screening and baseline survey to determine eligibility and demographic information, and a sleep study using full overnight polysomnography. An a priori power analysis was conducted to determine the minimum sample size needed to investigate OSA prevalence. An expected 75.7% prevalence of OSA in those with PTSD [3], and an OSA prevalence of 38% in the general population [21], indicated that a sample size of 52 participants would allow 80% power for *α* =.05. In order to account for 20% expected drop out, 63 individuals were recruited.

### Screening survey

Upon providing informed consent, participants completed a 30-minute survey hosted on an online platform (Qualtrics, https://www.qualtrics.com/) to assess trauma exposure, PTSD symptomatology, demographic factors and eligibility for polysomnography.

#### Posttraumatic stress disorder checklist (PCL-5)

PTSD symptomatology was assessed using the PCL-5 [22], a 20-item self-report measure relating to PTSD symptom clusters described in the DSM-5. Participants indicated the degree to which they experienced each symptom in the past month on a 5-point Likert scale ranging from 0 (not at all) to 4 (extremely). Responses were summed to a maximum possible score of 80. The PCL-5 has demonstrated robust internal consistency (*α* =.94) and test-retest reliability (*r* =.82) within university student samples [22] and a cut-off of between 31 and 33 was optimal for diagnosing PTSD [23, 24].

#### Life events checklist (LEC-5)

The LEC-5 [25] contains 16-items that examine exposure to potentially traumatic Criterion A events as per the Diagnostic and Statistical Manual of Mental Disorders (DSM-5), such as serious injury or natural disaster [26]. For each item, participants indicated whether they had experienced the traumatic event directly during their lifetime, or indirectly through witnessing, learning about or encountering the traumatic event second-hand. Selection of “happened to me” or “witnessed” for at least one event from items 1–16 was indicative of Criterion A trauma necessary for inclusion in the Likely and Subsyndromal PTSD groups. The LEC-5 has been validated in undergraduate student samples, demonstrating robust test-retest reliability (*r* =.82) [25].

#### Alcohol use disorder identification test (AUDIT)

The AUDIT [27] was included to screen for hazardous alcohol use, a potential confounding risk factor for OSA [28]. The AUDIT includes 10 items assessing drinking habits and frequency of alcohol-related problems on a 5-point Likert scale. Responses were summed to a maximum possible score of 40, with scores >15 considered hazardous alcohol consumption. The AUDIT has demonstrated sound psychometric properties in undergraduate student samples (internal consistency, *α* =.80 [29]).

#### Pittsburgh Sleep Quality Index (PSQI)

The PSQI [30] assesses sleep quality across seven domains via 19 items that participants rate on a 4-point Likert scale from 0 (no difficulty) to 3 (severe difficulty) over the past month. Responses were calculated to a maximum possible score of 21, with higher scores representing worse overall sleep quality with scores ≥5 indicating poor sleep quality. The PSQI has demonstrated robust internal consistency (*α* =.83 [31]) among undergraduate student samples.

#### Epworth sleepiness scale (ESS)

The ESS [32] consists of 8 items rated on a 4-point Likert scale ranging from 1 (no chance of dozing) to 3 (high chance of dozing). Higher scores indicate increased daytime sleepiness, and scores of ≥8 indicate substantial daytime sleepiness. The ESS is a well-validated measure used in both clinical and non-clinical samples [33] and has been shown to exhibit good internal consistency (*α* =.88).

#### Depression anxiety and stress scale (DASS-21)

The DASS-21 provides an overall assessment of general psychological distress over the past week across three domains—depression, anxiety, and stress [34]. It is a well-validated measure for use in non-clinical and clinical populations [35] and consists of 21 items rated on a 4-point Likert scale ranging from 0 (never) to 3 (almost always). Higher scores indicate elevated distress ranging from normal to extremely severe for each DASS scale. The DASS-21 has been shown to have excellent or good internal consistency across all subscales: depression (DASS-D, *α* =.91), anxiety (DASS-A, *α* =.84) and stress (DASS-S, *α* =.90) [34].

#### Insomnia severity index (ISI)

Items on the ISI examine insomnia symptoms following the International Classification of Sleep Disorders [36]. The ISI consists of 7 items rated on a 5-point Likert scale ranging from 0 to 4. Higher scores indicate greater insomnia severity with a global score of >8 indicating subthreshold insomnia symptoms [37]. The ISI has been shown to have excellent internal consistency (*α* =.91) [37].

#### Severity measure for panic disorder (PDSS)

This 10-item measure assesses the severity of panic disorder symptoms in adults over the last seven days. Each item is ranked on a 5-point Likert scale from 0 (never) to 4 (all of the time). Total scores range from 0 to 40 and a score of >8 indicates the presence of panic disorder [38]. The PDSS exhibits strong internal consistency and reliability in adults (*α* =.96 and.94) [39].

### Polysomnography eligibility criteria

Individuals with known sleep, respiratory or neurologic disorders or military experience were ineligible for the polysomnography study. Likewise, individuals aged <18 or > 65 years were excluded because OSA prevalence and causes differ in children and older adults. To minimise confounding, individuals indicating hazardous alcohol consumption and any level of illicit drug use were excluded [28]. Finally, any individuals reporting being pregnant, using testosterone, shiftwork or jetlag in the planned study period were not eligible to participate.

Probable PTSD severity was determined using participants’ global PCL-5 score alongside LEC-5 responses. Clinical interviews were not conducted to confirm diagnosis and so individuals with ambiguous PTSD symptoms (PCL-5 score < 15 with trauma re-experiencing symptoms; or PCL-5 score > 15 in the absence of trauma exposure) were excluded. Otherwise, participants were grouped as follows:

- Likely PTSD: Participants who experienced criterion A trauma, with PCL-5 scores of 33 or above that included re-experiencing symptoms (scoring ≥1 on PCL-5 items 1–5).
- Subsyndromal PTSD: Participants with PCL-5 scores between 15 and 33 that included re-experiencing symptoms and who also experienced Criterion A trauma exposure. This group was included to represent individuals with subclinical but relevant PTSD symptoms.
- No PTSD: Participants with PCL-5 scores below 15, without re-experiencing symptoms regardless of trauma exposure. Given the high lifetime prevalence (74.9%) of trauma exposure within the Australian population [40, 41], control participants were not required to be trauma naïve.

### Polysomnography

Participants underwent one night of standard polysomnography to detect respiratory events, enabling objective analysis of OSA prevalence. Studies were performed at home (Siesta, Compumedics, VIC, Australia) or in the laboratory (Grael, Compumedics, VIC, Australia) according to the participants’ preference. Identical procedures were followed regardless of where the participant slept and studies were conducted in accordance with the American Association of Sleep Medicine (AASM) guidelines [42]. The parameters measured included six electroencephalography (EEG) derivations, left and right electrooculography (EOG), submental electromyography (EMG), electrocardiography (ECG), left and right anterior tibialis EMG, chest and abdominal respiratory effort, finger oxygen saturation and body position. Thermistors were used for airflow assessment in the first 16 participants before nasal pressure recordings were also included for all subsequent participants. All data were acquired using Profusion PSG 3 software (Compumedics, VIC, Australia) with sampling rates of 512 Hz for EEG, EOG and EMG channels, 64 Hz for respiratory effort and nasal pressure, and 16 Hz for thermistor. Impedances were confirmed to be below 10 kΩ and bio-calibrations were performed before recording began. Participants were asked to go to bed at their usual bedtime and awaken at their normal rise time.

### Data analysis

A single qualified sleep technician, who was blind to participants’ PTSD status and demographic and anthropomorphic variables, conducted the scoring of sleep stages and respiratory events according to the AASM guidelines [42]. Apneas and hypopneas were identified when associated with either a 3% oxygen desaturation or arousal from sleep [42].

The ventilatory response to arousal was not calculated for participants with thermistor-based airflow assessments or those found to have OSA as increased resistance elevates arousal responses [43, 44]. For remaining participants, the ventilatory response to 3–15 s long arousals was estimated for each arousal that occurred from, and returned to, stable NREM sleep if the nasal pressure trace was adequate according to visual inspection (clear inspiration and expiration; no drift, clipping or artefact). The nasal pressure was integrated during each inspiration for the period 30 seconds before and 30 seconds after the arousal from sleep. In accordance with prior publications [45], if the arousal occurred during expiration, the next inspiration was considered the first breath of arousal. If the arousal occurred during the first half of an inspiration that breath was still considered the first breath of arousal. However, if the arousal occurred in the second half of an inspiration, the next inspiration was considered the first arousal breath. The estimated ventilation (integrated airflow during inspiration * respiratory rate) was calculated on a breath-by-breath basis and expressed as a percentage of the pre-arousal level (average of breaths −8 to −2 before the arousal breath) for each arousal.

### Statistical analyses

Analyses were conducted in RStudio (RStudio 2025.05.1 + 513, https://www.R-project.org/) with significance considered at *α* =.05. Assumptions for all statistical tests were examined and where violations occurred, non-parametric tests were used, or data were transformed. Kruskal-Wallis tests were conducted to determine whether the PTSD severity groups were similar in demographic characteristics relevant to OSA risk (i.e., age, sex, BMI, AUDIT score).

A Fisher’s exact test was employed to assess the first hypothesis that individuals with Likely PTSD would be more likely to demonstrate OSA. This test compared the counts of OSA positive and negative individuals across the PTSD groups (Likely, Subsyndromal and No PTSD). OSA was defined as an average of five or more respiratory events per hour of sleep (AHI ≥5). A Kruskal-Wallis rank sum test was used to compare AHI between groups as assumptions for analysis of variance were not met.

Multilevel modelling using lme4 package (version 1.1.35.1) with restricted maximum likelihood estimation was used for assessing the ventilatory response to arousal. This accounts for dependence between observations arising from repeated measures of arousal trials within the same participant by including a random intercept for trials nested within participants, and accommodates an unbalanced number of data points across individuals. The model compared the increase in ventilation across PTSD severity groups and included two fixed effects and their interactions: PTSD status and breath. Breath comprised 12 levels: pre-arousal breaths (−4 to −1), the first two arousal breaths (A1 and A2), and post-arousal breaths (1 to 6). Following previous literature, these breaths were included to allow a sufficient window to capture ventilatory changes associated with cortical arousal [44, 46]. Post-hoc analyses were undertaken using the emmeans package and pairwise comparisons were performed using Tukey’s test for multiple comparisons.

## Results

A total of 520 individuals completed the baseline survey from which 63 participants (19 Likely PTSD, 19 Subsyndromal PTSD and 25 No PTSD) were recruited for the polysomnography study. One Likely PTSD participant withdrew before the sleep study and the Siesta data card failed in 2 participants (one Likely PTSD and one No PTSD) leaving 60 participants with sleep study data. The demographics of the 60 included participants are shown in Table 1. The groups did not differ in the proportion of female participants, age or body mass index (BMI). However, all three groups differed on PCL-5 (by design), ISI, DASS-D, DASS-A, DASS-S and PDSS scores with psychopathology increasing by PTSD severity (all *p* <.001). Likewise, PSQI (*p* =.006) and ESS (*p* <.001) were lower in No PTSD group than Likely and Subsyndromal PTSD groups, who did not differ from each other. Individual items on the ISI were assessed in order to examine insomnia differences more carefully, and all seven items differed between No PTSD and Likely PTSD groups, with the subsyndromal values falling in the middle and not differing to either group statistically.

**Table 1:** Participant demographics and questionnaire results in PTSD groups

	Full sample	Likely-PTSD	Sub-PTSD	No PTSD
N (Female)	60 (42)	18 (11)	19 (14)	23 (17)
BMI (kg/m^2^)	23.7 ± 4.8	25.1 ± 5.4	22.3 ± 4.1	23.9 ± 3.4
Age (years)	24.9 ± 6.3	24.3 ± 6.4	23.9 ± 4.3	26.2 ± 7.5
PCL-5 *	22.9 ± 18.6	46.2 ± 9.8	23.6 ± 4.1	4.0 ± 4.4
PSQI	6.3 ± 2.5	7.6 ± 2.5 ^#^	6.5 ± 1.9 ^#^	4.8 ± 2.4
ESS	6.4 ± 4.7	8.6 ± 5.5 ^#^	7.7 ± 4.0 ^#^	3.4 ± 2.9
DASS-D *	5.6 ± 4.9	9.9 ± 4.9	6.1 ± 3.5	1.8 ± 2.3
DASS-A *	4.5 ± 4.1	8.2 ± 4.0	4.9 ± 2.6	1.3 ± 1.9
DASS-S *	7.2 ± 4.4	11.6 ± 3.6	7.0 ± 2.1	3.9 ± 3.6
ISI *	9.5 ± 5.6	13.8 ± 4.4	9.6 ± 4.3	5.1 ± 4.7
PDSS *	0.5 ± 0.7	1.2 ± 0.8	0.3 ± 0.4	0.1 ± 0.2

Mean ± SD *BMI* body mass index, *PCL-5* Posttraumatic Stress Disorder Checklist, *PSQI* Pittsburgh Sleep Quality Index, *ESS* Epworth Sleepiness Scale, *D* Depression, *A* Anxiety and *S* Stress, *DASS* Score, *ISI* Insomnia Severity Index, *PDSS* panic disorder severity measure. * significant difference between all 3 groups, ^#^ significant difference to No PTSD group

**Fig. 1:**
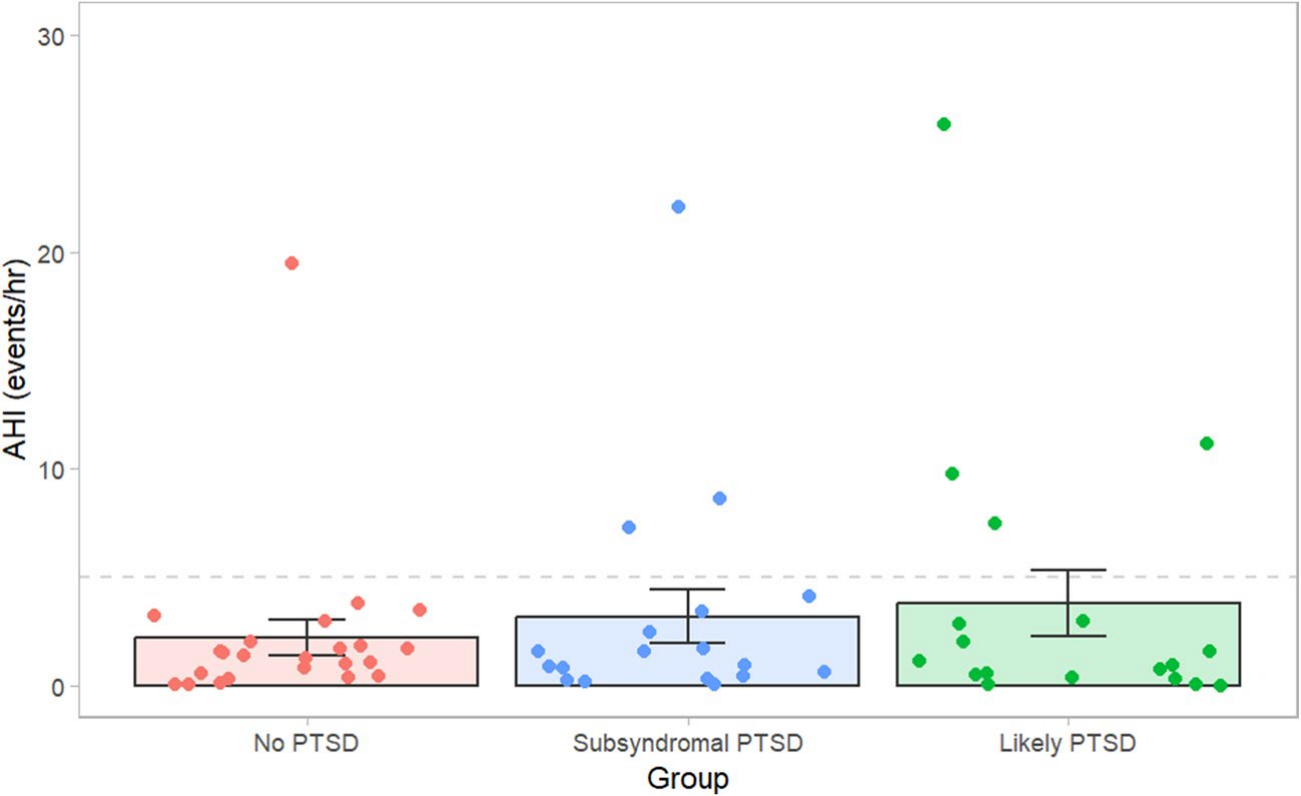
Individual participant (data points) and group mean (±SEM) AHI in No PTSD, Subsyndromal PTSD and Likely PTSD groups. No statistically significant difference in AHI was found between groups (*p* = 0.98), *n* = 60

Obstructive sleep apnea was observed in 8 participants, but the proportion did not significantly differ between groups (*p* =.212) with 4 Likely PTSD, 3 Subsyndromal PTSD and 1 No PTSD individuals having AHI > 5 events per hour. Although not significantly different, the odds of having OSA appeared higher in Likely PTSD (OR = 6.02, 95% CI: 0.53–322.66, *p* = 0.15) and Subsyndromal PTSD groups (OR = 3.99. 95% CI: 0.29–225.87.29.87, *p* =.24). Likewise, a Kruskal-Wallis rank sum test showed the AHI did not differ between PTSD status groups (Fig. 1 and Table 2, χ^2^(2) = 0.040, *p* =.98). Other sleep study data are reported in Table 2.

**Table 2:** Polysomnography data in PTSD groups

	Likely-PTSD	Sub-PTSD	No PTSD
AHI (events/hr)	3.8 ± 6.5	3.2 ± 5.3	2.2 ± 3.9
TST (min)	367.5 ± 117.9	335.2 ± 127.9	406.3 ± 61.6
SE (%)	76.9 ± 22.1	75.8 ± 18.9	86.2 ± 8.3
SOL (min)	47.6 ± 58.5 ^#^	64.2 ± 93.8 ^#^	24.5 ± 35.9
WASO (min)	38.5 ± 35.9	31.8 ± 27.8	39.9 ± 17.1
REM Latency (min)	98.9 ± 45.6	98.2 ± 40.4	104.6 ± 47.4
N1 (min)	28.4 ± 16.2	23.5 ± 14.3	32.9 ± 14.8
N2 (min)	167.1 ± 72.9	159.0 ± 62.2	183.2 ± 43.2
N3 (min)	88.3 ± 30.0	84.0 ± 30.7	96.9 ± 32.2
REM (min)	83.6 ± 49.3	68.7 ± 49.2	91.0 ± 25.5
Spont ArI (/hr)	7.2 ± 2.4	7.1 ± 2.9	9.9 ± 3.9
Resp ArI (/hr)	3.1 ± 5.5	2.2 ± 5.1	1.3 ± 2.7
Lowest SpO_2_ (%)	87.9 ± 5.1	91.0 ± 4.4	89.0 ± 5.5

Mean and standard deviation of polysomnography data in the 3 PTSD groups. *AHI* apnea-hypopnea index, *TST* total sleep time, *SE* sleep efficiency, *SOL* sleep onset latency, *WASO* wake after sleep onset, *REM latentcy* the latency to first REM sleep, *N1* duration of stage 1, *N2* duration of stage 2, *N3* slow wave sleep duration, *REM* duration of REM sleep, *Spont ArI* spontaneous arousal index, *Resp ArI* respiratory arousal index, *lowest SpO2* lowest oxygen saturation. ^#^ indicates a significant difference to the No PTSD group

The ventilatory response to arousal from sleep could be calculated in 29 participants (10 with Likely PTSD, 12 Subsyndromal PTSD and 7 with No PTSD). Reasons for not including the other participants include having OSA (*n* = 8), having airflow assessed with thermistor only (*n* = 16), no brief arousals arising from and returning to stable sleep (*n* = 2), and poor nasal pressure signal quality (*n* = 5). In the 29 participants with data, a total of 232 arousals were analysed (66 in Likely PTSD, 67 in Subsyndromal PTSD and 99 in No PTSD). The average duration of arousal did not differ between groups (6.3 ± 2.6 s in Likely PTSD, 6.3 ± 2.7 s in Subsyndromal and 5.6 ± 2.2 s in No PTSD, χ^2^(2) = 3.46, *p* =.18).

**Fig. 2:**
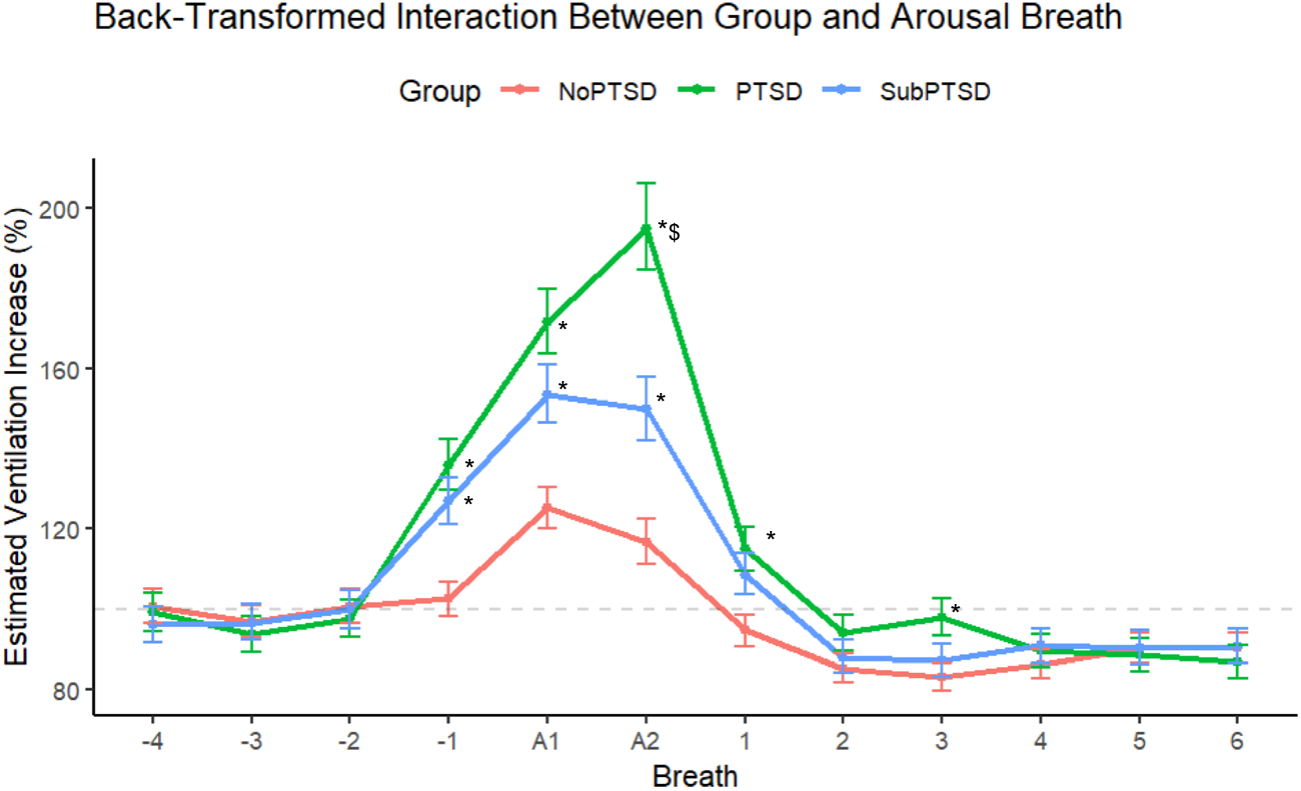
Back-transformed estimated ventilatory response to arousal according to PTSD grouping. Breaths −4 to −1 are the four breaths before arousal from sleep, breaths A1 and A2 are the first two breaths during arousal and Breaths 1–6 are the six breaths following the return to sleep after arousal. * indicates different to No PTSD on that breath, ^$^ indicates different to Subsyndromal PTSD on that breath. Main effects of group (*p* =.016), breath (*p* <.001), and the interaction (*p* <.001) were all significant, *n* = 29

Estimated ventilation was log transformed due to non-normally distributed data before being analysed with a multilevel model. Group F(2, 17.34) = 5.25, *p* =.016, Breath F(11, 2444.75) = 69.52, *p* <.001 and their interaction F(22, 2444.76) = 5.15, *p* <.001 were all significant. The group effect occurred because Likely PTSD differed from No PTSD, with subsyndromal PTSD not differing from either group. The interaction effect occurred because both Likely PTSD and Subsyndromal groups differed to No PTSD on the last breath prior to arousal and during the arousal (−1, A1 and A2), with the Likely PTSD group being higher than Subsyndromal PTSD on the second breath of arousal (A2). The Likely PTSD group was also higher than the No PTSD on the first and third breaths following the return to sleep (1 and 3, see Fig. 2).

## Discussion

The aims of this paper were to determine whether young adults with PTSD symptoms resulting from non-military trauma exposure had elevated rates of unrecognised OSA, and to compare the ventilatory response to brief spontaneous arousal from sleep between groups with differing PTSD symptom levels. In contrast to numerous studies conducted in middle-aged, predominantly male veterans [3], we did not see a statistically increased occurrence of OSA in young individuals displaying PTSD symptoms, although the proportions (22.2% in Likely PTSD, 15.8% in Subsyndromal PTSD and 4.3% in No PTSD) and odds ratios tended to be elevated. It is possible that the current study was underpowered and a true difference in OSA prevalence exists even in this predominantly young non-obese population. In order to detect a difference of the magnitude observed in this study with *α* = 0.05 and 80% power, approximately 66 people would be required in each group. Future larger studies, potentially employing simpler OSA detection methods that can record over more than 1 night, may be useful to further investigate this question, as single night studies have been estimated to misclassify patients 21% of the time [47]. Alternatively, the prevalence of OSA may not be elevated in young, non-overweight individuals with mixed, non-military trauma histories. If this is the case then the high prevalence of OSA observed in prior studies may relate to the characteristics of the veterans studied such as the predominance of men, high body weight and high rates of alcohol use. Future research is required to separate these possibilities.

The sleep architecture of the young individuals studied also did not differ greatly between those with and without PTSD, with only sleep onset latency being significantly elevated in both likely and subsyndromal PTSD groups compared to individuals without PTSD. This is in contrast to some prior studies that have found that individuals with PTSD have lower total sleep times, reduced sleep efficiency, increased wake after sleep onset and reduced N3 amount [48]. However, the magnitude of differences were largely as expected, perhaps with the exception of sleep onset latency and REM latency which were relatively high in the current study. Despite not finding high rates of OSA and similar sleep architecture between groups, the ventilatory response to brief spontaneous arousal from sleep was elevated in individuals with likely PTSD. Prior studies investigating the ventilatory response to arousal have shown that responses are increased when resistance during sleep prior to the arousal is elevated [43, 44]. Thus, even though participants with OSA were excluded from this analysis, some people with elevated upper airway resistance may have remained in the PTSD groups and would be expected to have elevated ventilatory responses. However, clear snoring or flow limitation was not commonly observed making this appear unlikely. In the current study, the ventilatory response to arousal was combined in N2 and N3 sleep. Sleep stage-related differences in airway resistance could theoretically result in the ventilatory response to arousal differing between sleep stages. However, in a prior study in healthy young individuals without PTSD the ventilatory response to arousal did not differ between N3 and N2 [46]. Thus, we cannot be sure whether the combination of sleep stages influenced our findings without further direct comparison. Another consideration is that men have been shown to have higher ventilatory responses to arousal than women [46]. Overall, the current study had a majority of female participants and of the 29 individuals who contributed to the ventilatory response to arousal data, only 8 were male. However, the proportion of male participants did tend to be lower in the No PTSD group (1 of 7) than in Likely PTSD (3 of 7) and Subsyndromal PTSD (4 of 8) groups. On the other hand, the prior paper reporting sex differences in healthy individuals had mean increases of 10–20% above the stable sleeping level [46] - far smaller than the increases in ventilation observed at arousal in the current study. Thus, further research is required to determine whether the sex of the participants contributes to the ventilatory response to arousal in PTSD.

### Clinical implications

Recent studies have demonstrated that the ventilatory response to arousal can be lowered in patients with OSA with acetazolamide [49] or venlafaxine [15] with concomitant reductions in the severity of OSA observed [50, 51]. This leaves open the possibility that if the ventilatory response to arousal contributes to the high prevalence of OSA in PTSD, then therapeutic manipulation may be feasible. Interestingly, acetazolamide also has been shown to have beneficial fear memory consolidation effects in rats [52], which if present in humans with PTSD may have additional benefit as fear memory processing is altered in PTSD and thought to contribute to the condition [53].

Alternate methods of lowering the ventilatory response to arousal could also be considered. For example, if the elevated ventilatory response to arousal in PTSD is a result of hyperarousal, cognitive behavioural therapy for insomnia (CBTi) may have utility in lowering it. Certainly, in patients with comorbid insomnia and obstructive sleep apnea, CBTi alone can lower the AHI providing some support for this concept [54, 55]. However, the biological mechanisms leading to OSA (OSA endotypes) were not altered following CBTi, although it should be noted that the ventilatory response to arousal was not assessed in this study [55]. Likewise, it is possible that the elevated ventilatory response to arousal reflects altered interoception in people with PTSD [12]. If so, it may follow that techniques to alter interoception such as mindfulness and slow breathing techniques [56] may also influence the magnitude of the ventilatory response to arousal. Future research is required to investigate these possibilities.

Another consideration regarding the elevated ventilatory response to arousal in PTSD is that it may contribute to the overscoring of respiratory events in PTSD. The severity of a respiratory event may appear greater if the increase in ventilation at the time of arousal is elevated. While technicians are instructed to score events as a reduction from stable ventilation levels [57], the period of stable ventilation may have been some time earlier in the file. This is a theoretical consideration that requires further research in PTSD populations to determine whether overscoring plays a role in OSA diagnosis.

### Limitations

Despite the current study’s strength of recruiting a diverse population of individuals with non-military trauma exposure, it has some limitations. Firstly, as noted above, the finding of OSA prevalence being similar between groups may be due to inadequate statistical power. Seven of eight participants with OSA endorsed some level of PTSD symptoms, including re-experiencing symptoms. Larger future studies will be required to investigate OSA prevalence further. Secondly, our sample represents a population at an already low risk of having OSA, being that they were predominantly young and with low BMIs. Of note, prior studies in individuals with non-military traumas have reported high rates of OSA in sexual assault survivors [7] or victims of crime [6] with only slightly older age and higher BMI than our sample. While this supports the choice to investigate OSA prevalence in the young population, we are nonetheless limited in our ability to measure how risk factors such as age and weight might interact with the observed heightened VRA to predispose OSA in the PTSD population. Thirdly, we did not perform clinical diagnostic interviews to diagnose PTSD. However, we used stringent cutoffs for considering likely or subsyndromal PTSD with the requirement that they must have elevated PCL-5 scores in addition to endorsing re-experiencing symptoms (nightmares or intrusions). Finally, we have estimated the ventilatory response to arousal using nasal pressure as opposed to the gold standard pneumotachograph-derived ventilation measures. While it is extremely common to use nasal pressure to approximate ventilation in phenotyping studies [10, 58] and prior studies assessing the ventilatory response to arousal have also analysed nasal pressure in this way [15, 49, 50], it remains possible that different results would be obtained with gold-standard measurements. Finally, only a portion of participants had data available for ventilatory response to arousal calculation, and as such the numbers in each subgroup were low. However, the number of arousals included was quite large at over 200 reducing variability. Ultimately further replication in larger, clinical populations with larger samples will be required to confirm the observation of elevated ventilatory response to arousal from sleep in people with PTSD symptoms.

### Conclusion

In conclusion, the prevalence of OSA in young, normal-weight individuals with non-military trauma exposure appears lower than prior studies in military veterans. However, the ventilatory response to arousal from sleep was elevated in this population. If an elevated ventilatory response to arousal persists in individuals with PTSD and other OSA risk factors such as older age and higher BMI, it could lead to high rates of OSA. Importantly, the ventilatory response to arousal can be lowered pharmacologically in OSA with subsequent reductions in OSA severity observed [15, 49, 50]. Thus, lowering the ventilatory responses to arousal in PTSD may be a personalised treatment target. This is important because CPAP tolerance and adherence is worse in PTSD than in the general OSA population [20], and poor sleep contributes to the poor outcomes in PTSD. Thus, successful treatment of OSA in PTSD has a strong potential for improving both the high personal and societal costs of PTSD and OSA.

## Data Availability

The data underlying this article cannot be shared publicly because extended consent was obtained from participants, such that the data can only be used for this project or closely related projects. The data will be shared on reasonable request to the corresponding author if this criteria is met.
